# Suicide in the Elderly – A Prevalent Phenomenon With Low Societal Awareness

**DOI:** 10.3389/ijph.2024.1606482

**Published:** 2024-02-21

**Authors:** Sophia Werdin

**Affiliations:** ^1^ Swiss Tropical and Public Health Institute, Allschwil, Switzerland; ^2^ University of Basel, Basel, Switzerland

**Keywords:** suicide, older people, suicide prevention, awareness, mental health

**The IJPH series “Young Researcher Editorial” is a training project of the Swiss School of Public Health.**

Suicide in youths attracts public attention, inspires deep sorrow, and is met with incomprehension. However, suicides in the elderly are rarely discussed. This editorial focuses on suicides among individuals aged 65 and older. High-income countries have introduced suicide prevention programs for adolescents and adults, but they do not often focus on the elderly. Furthermore, research on suicidality in older people, its risk factors, and prevention is scarce [1] despite the high suicide rate in this population.

Data from most countries show that suicide rates peak in old age [2]. For example, among all age groups in Switzerland in 2021, 13.9 men and 5.5 women out of every 100,000 committed suicide [3]. In contrast, the rate in the 85+ age group was 56.3 men and 11.2 women per 100,000 [3]. In addition to registered suicides, we must assume a substantial, but barely quantified, number of unreported suicides and suicide attempts. Numerous incidents remain hidden due to the stigma surrounding suicidal thoughts and are therefore not included in any statistics [2]. Furthermore, not all suicides are recorded as such. For instance, it is unclear how frequently death certificates of elderly individuals indicate natural death instead of suicide due to physicians trying to mitigate the burden on relatives [2, 4]. Besides this, there are several challenges in recognizing suicides among older individuals during medical postmortem examinations. These include the absence of external injuries and signs of difficult-to-trace, so-called “soft” suicide methods (e.g., poisoning), degradation of the corpse as a result of being left for a long period, as well as intentional or negligent alteration of the scene when the corpse is found by third parties [4]. The proportion of incorrectly certified deaths is likely to increase with age as the older the victim is, the more likely it is that natural death will be assumed [5].

Even though assisted suicides are usually published separately from suicide statistics and are often categorized differently [6], it is important to note that it is mainly older people that choose this approach to deliberately end their life. In assisted suicide, suicide-willing individuals are given a lethal substance which they choose to ingest themselves without outside intervention [6]. Switzerland is one of the few countries where this practice is legally permitted. The decision to commit assisted suicide is commonly linked to a serious, incurable illness [6]. In 2021, statistics from Switzerland showed that the number of assisted suicides by residents aged 65 and older was 3.5 times higher than the number of reported suicides in the same age group [3]. Over a decade, there was an almost 350% increase in assisted suicides within this demographic [3]. Statistics on reported suicides, combined with a potentially high number of unreported cases and data on assisted suicides, suggest that many older individuals suffer profound grief.

Getting older increases the risk of various health conditions and stressors that can interact in complex ways to cause suicidal behavior. For example, psychiatric and neurocognitive disorders (e.g., depression), social isolation, physical impairments (e.g., cancer, pain), and life events, such as the loss of a spouse, are associated with suicidal ideation [1, 7]. However, symptoms of mental illnesses and behavioral changes are often overlooked or attributed to the aging process [8]. Older suicidal individuals may have difficulty accessing mental healthcare due to limited mobility, isolated living with small social networks, and lack of digital literacy [9]. Furthermore, diagnosed mental illnesses in elderly individuals may not receive adequate treatment [8], which could be attributed to factors such as a shortage of geriatric mental health specialists and comorbidities, making psychological issues less of a priority.

There are opportunities to lower the suicide rate among the elderly. Since 58% of individuals aged 55 or older visit their primary care provider in the month before they commit suicide [10], general medical practitioners should be trained in recognizing suicidal ideation [7]. The elderly could also benefit from targeted suicide prevention programs that address age-related risk factors and meet their needs for psychological counseling or treatment. Mobile outreach approaches like *ASSIP Home Treatment* [11] hold promise, providing the established *Attempted Suicide Short Intervention Program* (*ASSIP*) to patient’s homes. *ASSIP Home Treatment* surmounts a major access barrier and helps previously hard-to-reach patients cope with suicidal crises.

Focused public health efforts are required to address suicide risk among the elderly. Their specific risk factors and consistently high suicide rate highlight the urgency of developing and implementing tailored prevention programs. We must raise awareness of older people’s mental health in both healthcare settings and the general population, and support mental health capabilities of the elderly to reduce the number of suicides in this vulnerable and high-risk population.

## References

[1] Lapierre S Erlangsen A Waern M De Leo D Oyama H Scocco P A Systematic Review of Elderly Suicide Prevention Programs. Crisis (2011) 32(2):88–98. 10.1027/0227-5910/a000076 21602163 PMC3728773

[2] World Health Organization. Suicide Worldwide in 2019: Global Health Estimates. Geneva: World Health Organization (2021).

[3] FSO. Causes of Death Statistics. Neuchâtel, Switzerland: Swiss Federal Statistical Office (2023). cited 11/08/2023.

[4] Madea B. Strukturelle Probleme Bei Der Leichenschau. Rechtsmedizin (2009) 19(6):399–406. 10.1007/s00194-009-0638-8

[5] Jackowski C. Der Plötzliche Natürliche Tod Skriptum Rechtsmedizin. Bern: University of Bern and University of Lucerne (2023).

[6] Junker C. Assistierter Suizid (Sterbehilfe) und Suizid in der Schweiz. Neuchâtel: Federal Statistical Office (2016).

[7] Conejero I Olié E Courtet P Calati R. Suicide in Older Adults: Current Perspectives. Clin Interv Aging (2018) 13:691–9. 10.2147/CIA.S130670 29719381 PMC5916258

[8] Voros V Fekete S Tenyi T Rihmer Z Szili I Osvath P. Untreated Depressive Symptoms Significantly Worsen Quality of Life in Old Age and May Lead to the Misdiagnosis of Dementia: A Cross-Sectional Study. Ann Gen Psychiatry (2020) 19(1):52. 10.1186/s12991-020-00302-6 32944058 PMC7493324

[9] Reynolds CF 3rd Jeste DV Sachdev PS Blazer DG. Mental Health Care for Older Adults: Recent Advances and New Directions in Clinical Practice and Research. World Psychiatry (2022) 21(3):336–63. 10.1002/wps.20996 36073714 PMC9453913

[10] Luoma JB Martin CE Pearson JL. Contact With Mental Health and Primary Care Providers Before Suicide: A Review of the Evidence. Am J Psychiatry (2002) 159(6):909–16. 10.1176/appi.ajp.159.6.909 12042175 PMC5072576

[11] University Psychiatric Services Bern AG. Was ist ASSIP Home Treatment? (2022). Available from: https://www.assip.org/fuer-patientinnen/was-ist-assip-home-treatment-2/ (Accessed February 7, 2024).

